# Isolation and characterization of two novel bacteriophages against carbapenem-resistant *Klebsiella pneumoniae*

**DOI:** 10.3389/fcimb.2024.1421724

**Published:** 2024-08-29

**Authors:** Abrar Senhaji-Kacha, Mireia Bernabéu-Gimeno, Pilar Domingo-Calap, John Jairo Aguilera-Correa, Mateo Seoane-Blanco, Sara Otaegi-Ugartemendia, Mark J. van Raaij, Jaime Esteban, Meritxell García-Quintanilla

**Affiliations:** ^1^ Department of Clinical Microbiology, Health Research Institute or Instituto de Investigación Sanitaria (IIS)-Fundación Jiménez Díaz, Universidad Autónoma de Madrid (UAM), Madrid, Spain; ^2^ CIBERINFEC-CIBER of Infectious Diseases, Madrid, Spain; ^3^ Institute of Biología Integrativa de Sistemas, Universitat de València-The Spanish National Research Council or Consejo Superior de Investigaciones Científicas (CSIC), Paterna, Spain; ^4^ Department of Macromolecular Structure, Centro Nacional de Biotecnología-The Spanish National Research Council or Consejo Superior de Investigaciones Científicas (CNB-CSIC), Madrid, Spain

**Keywords:** bacteriophages, phage therapy, *Klebsiella pneumoniae*, multidrug-resistant (MDR), alternative therapy

## Abstract

The increase of antibiotic-resistant bacteria has become a global health emergency and the need to explore alternative therapeutic options arises. Phage therapy uses bacteriophages to target specific bacterial strains. Phages are highly specific and can target resistant bacteria. Currently, research in this regard is focused on ensuring reliability and safety to bring this tool into clinical practice. The first step is to conduct comprehensive preclinical research. In this work, we present two novel bacteriophages vB_Kpn_F13 and vB_Kpn_F14 isolated against clinical carbapenem-resistant *Klebsiella pneumoniae* strains obtained from hospital sewage. Multiple studies *in vitro* were conducted, such as sequencing, electron microscopy, stability, host range infectivity, planktonic effect and biofilm inhibition in order to discover their ability to be used against carbapenem-resistant *K. pneumoniae* pathogens causing difficult-to-treat infections.

## 1 Introduction

*K. pneumoniae* is an opportunistic pathogen capable of colonizing mucosal surfaces without causing disease. However, it may disseminate to other tissues, causing life-threatening infections including pneumonia, urinary tract infections, bloodstream infections and sepsis (Ashurst and Dawson, 2023). *K. pneumoniae* is also responsible for a significant number of community-acquired infections world-wide (Ko et al., 2002). Some of the *Klebsiella* strains associated with these infections are considered as hypervirulent (hvKP), due to their enhanced virulent factors (siderophores, specific polysaccharide capsule serotypes, *rmpA* genes), and recent epidemiological studies indicate that they share specific genetic characteristics (Holt et al., 2015).

In Europe, *K*. *pneumoniae* carbapenemase (KPC) is the most prevalent carbapenemase gene in *K*. *pneumoniae* nosocomial infections (45%), followed by oxacillinase-48 (OXA-48-like) (37%) (Mohammadi et al., 2023). This bacterium is also capable of developing biofilm, which can diminish the penetration of antibiotics and host immune cells, among other defense mechanisms (Deva et al., 2013).

Treatment options against carbapenem-resistant *K. pneumoniae* are limited to a few antibiotics (a carbapenem e.g. meropenem combined with colistin and/or tigecycline, gentamicin or fosfomycin), though resistance has also been described exceeding 50% in parts of the Eastern Mediterranean and Europe, unfortunately (Zhu et al., 2020). In 2014, the US National Institute of Allergy and Infectious Diseases (NIAID) included phage therapy as one of seven strategies to tackle antibiotic resistance (Reardon, 2014). Bacteriophages are viruses able to infect bacteria. They are ubiquitous biological entities found in nature with highly diverse genetic compositions and were first discovered in 1917 by Felix d’Herelle. However, the first suspicion of the existence of a bacteriolytic agent was made by Frederick Twort (Gordillo Altamirano and Barr, 2019). Depending on the biological cycle, lytic or lysogenic, bacteriophages can be classified as virulent or temperate, respectively (Kasman and Porter, 2023). Virulent phages are most desirable for therapeutic use mostly to avoid potential transfer of bacterial DNA, including antibiotic resistance or virulence genes, from temperate phages. Phages can also express polysaccharide depolymerases and endolysins, which degrade extracellular polysaccharides or peptidoglycan, respectively, and disrupt biofilm structures (Ghose and Euler, 2020).

There is an unmet clinical need of finding reliable bacteriophage candidates able to inhibit the growth of clinical strains of *K. pneumoniae* (including carbapenem-resistant strains), both in liquid and in mature biofilms.

This study characterizes and analyzes the *in vitro* activity of two novel bacteriophages, vB_Kpn_F13 and vB_Kpn_F14 (referred to as F13 and F14, respectively), isolated *de novo* against carbapenem-resistant *K. pneumoniae* clinical strains in planktonic and biofilm state.

## 2 Materials and methods

### 2.1 Bacterial strains and growth conditions

*K. pneumoniae* ATCC 23357, a pandrug-resistant strain and 46 clinical isolates (**Supplementary Table 1**) were used for the determination of host range. This study uses strains obtained and kindly donated from the Microbiology Department of the Hospital Universitario Fundación Jimenez Díaz (HUFJD). The Research Ethics Committee of the Health Research Institute Fundación Jiménez Díaz did not require the study to be reviewed or approved by an ethics committee because the researchers just received the clinical strains already isolated, not the human samples, and no information from the patient´s medical history was needed for this work. Isolates were stored in sterile skimmed milk (Difco™, USA) at −20°C. Strains were plated from frozen stocks onto Mueller Hinton E agar (MHE) and tryptic soy broth (TSB) media (BioMérieux, France). Agar plates and broth cultures were incubated at 37°C overnight.

### 2.2 Determination of genomic sequence type (ST) and *wzi* sequencing

Strains infected by the two phages were analyzed by MLST according to the Pasteur webpage instructions (http://www.pasteur.fr/mlst).

K-type variation is linked to the *cps* locus, which has a mosaic structure at its 5’ end with a group of six conserved genes (*galF*, *orf2*, *wzi*, *wza*, *wzb*, and *wzc*). The *wzi* gene is conserved in all capsular types of *K. pneumoniae*. Only susceptible strains were subjected to analysis. The *wzi* sequencing was performed according to a previously published protocol (Brisse et al., 2013).

Capsular determination serotype for *K. pneumoniae* clinical strains forward and reverse chromatograms were visualized and edited using *Chromas version 2.6.6* and assembled with *Pregap4 version 1.6* and *Gap version 4.11.* K-PAM *in silico* diagnostic tool was supposed to be used for the prediction of the capsular type from the FASTA file obtained for each isolate. However, due to technical problems in the webpage, Wzi reference data base from *Kaptive* and three different **Supplementary Data** sets were reviewed and compared with our results to confirm identity above 99,5% and finally determine the capsular type of the clinical isolates. Links to these data bases can be found at *References* section.

### 2.3 Isolation, propagation and titration of bacteriophages

Bacteriophages were obtained from the HUFJD sewage (Madrid, Spain). Liquid samples were centrifuged (12,000 x g, 10 min, 4°C) for removing fecal matter. The supernatant was filtered using 0.45 µm and 0.22 µm PES syringe filters to remove all the bacteria and other small-size debris. For enrichment, 100 µl of bacteria and 10 ml of filtered supernatant from sewage were mixed with 10 ml of 2x TSB and incubated overnight, and 200 µl of the enriched mix was plated using the double-layer agar method (Kropinski et al., 2009) with a top agar containing 0.2% (V/W) of agarose (PanreacQuímica, USA). Formation of plaques confirmed the presence of phages.

For the isolation, individual plaques with different morphology were picked using sterile Pasteur pipettes (ThermoFisher Scientific, USA) and placed into 1.5 ml Eppendorf tubes (Eppendorf, Germany) containing 1 ml of sodium magnesium (SM) buffer (SM; 100 mM sodium chloride; 10 mM magnesium sulfate; 10 mM calcium chloride; 50 mM Tris HCl, pH 7.5), and vortexed vigorously for 1 min. The collected samples were then centrifuged, and the supernatant was collected and cultured using the double-layer agar method. This procedure was performed three times to ensure the purification of a single phage type.

For phage amplification, KP5 in exponential phase and phages were added into TSB containing 10 mM MgSO_4_ and 10 mM CaCl_2_ and incubated for 4–5 h at 37°C 200 rpm shaking. The supernatant was harvested and filtrated with 0.45 µm and 0.22 µm filters (Luong et al., 2020; Camens et al., 2021). Phage titer was determined via a double-layer agar method.

### 2.4 Thermal and pH stability

A working stock in TSB contained an initial phage titer of 10^8^ PFU/ml. For pH test, 100 µl of each phage was suspended in 1 ml SM buffer previously adjusted to pH 1, 4.5, 7.4 and 8. Samples were incubated at room temperature for 1 h and the titer of phages was determined. For thermal testing, 100 µl of the working stock phage was suspended in 1 ml of SM buffer and incubated at -80°C, -20°C, 4°C, 21°C, 37°C, and 60°C for 1 h, 24 h and 168 h (Bae et al., 2012; Senhaji-Kacha et al., 2020).

### 2.5 Determination of host range

The lytic activity of F13 and F14 was determined against 47 clinical carbapenem-resistant *K. pneumoniae* strains using spot testing (Shi et al., 2020). Briefly, 5 μl of the purified phage suspension (10^8^ PFU/ml) and serial dilutions of each phage (1:1, 1:10, 1:100) were spotted onto the surface of TSB double-layer agar plate previously inoculated with the tested strains. After adsorption of phage suspension, plates were incubated overnight at 37°C. Host range was determined by plaque formation.

### 2.6 One-step growth curve

Phage latent period and burst size were determined by a one-step growth experiment according to the methodology described previously (Zurabov and Zhilenkov, 2021), with minor modifications. In 1 ml of TSB broth, a KP5 suspension of 10^8^ CFU/ml and a phage filtrate of 10^6^ PFU/ml were mixed. Mixture was incubated at 37°C for 8 min static and then centrifuged for 10 min at 16.000 x g. Supernatant was removed, the pellet was resuspended in 100 ml of TSB and the final suspension was incubated at 37°C with 180 rpm. Aliquots of 500 μl were taken every 10 min for 120 min and the bacteriophage titre was assessed using the double-layer agar method. Plaques were counted after overnight incubation at 37°C. The latent period is the interval between adsorption and host lysis. Burst size is the average number of phage virions released from infected bacterial cells and was calculate dividing the maximum phage yield between the initial phage yield.

### 2.7 Transmission electron microscopy

In order to determine the family and morphology of the isolated phages, transmission electron microscopy (TEM) was performed. Briefly, carbon/colloid coated copper grids (Gilder Grids) with the purified and high titer phage (10^9^ PFU/ml) stocks were negatively stained with 2% (w/v) uranyl acetate. Images of the sample were taken in a 100 kV JEOL JEM 1011 transmission electron microscope (JEOL). Measurements of phage dimensions were done with Fiji (Schindelin et al., 2012).

### 2.8 Bacteriophage sequencing and in silico genomic analysis of phages

For DNA extraction, 10 µl of high-titer lysates were treated with DNAse I, capsids were digested with Proteinase K and phage DNA were purified using DNA Clean & Concentrator 5 kit (Zymo). The library, prepared using the Nextera XT Library prep kit, was used to perform sequencing using Illumina MiSeq technology (150 bp paired-end reads). Quality control of the reads was performed with fastp (min_length: 50, trim_qual_right: 30, trim_qual_type: mean and trim_qual_window: 10) (Chen et al., 2018) and the resulting reads were used to perform a *de novo* assembly with SPAdes-3.15.4 (only-assembler mode) (Bankevich et al., 2012). The contigs were analyzed and those of low length and coverage and those coming from the bacterial host were discarded. Once removed, the contig with high length and k-mer coverage per sample was taken as the phage genome and compared with BLAST (Altschul et al., 1990) against the nucleotide database to find the closest relative of each genome.

As the packaging strategy of phages could not be predicted given the method of library preparation using transposases, the large terminase subunit was used as the first gene to rearrange the genomes. The rearranged genomes were corrected with Pilon (Walker et al., 2014) and the coverage was evaluated by mapping all the cleaned reads with bbmap.sh (Bushnell, 2014). Genomes were further analyzed using PhageLeads (Yukgehnaish et al., 2022) to predict phage temperate lifestyle, virulence and antimicrobial resistance genes. Structural and functional annotation of the genomes was performed using multiphate2 (Ecale Zhou et al., 2021). For this, Glimmer (Delcher et al., 1999), Prodigal (Hyatt et al., 2010) and Phanotate (McNair et al., 2019) were used to generate a consensus gene call that was functionally annotated by BLAST (Altschul et al., 1990) against RefSeq protein database (minimum identity of 60%). Additionally, a profile-to-sequence comparison was performed using mmseqs (Hauser et al., 2016) and phrogs profile db (Terzian et al., 2021) (sensitivity set to 7). Each coding sequence was associated to a functional group to facilitate representation. Both genomes were compared by BLAST and represented with gggenomes software (A grammar of graphics for comparative genomics, 2024).

Additional comparative analyses were performed to have a further understanding of the relationship between our phages and previously described ones. Firstly, a single-gene phylogeny using the amino acid sequence of the tail tape measure protein was performed using ClustalW (Thompson et al., 1994) for the alignment and IQ-TREE (Trifinopoulos et al., 2016) to construct a maximum likelihood phylogeny with 1000 fast bootstrap pseudo replicates that was represented using iTOL (Letunic and Bork, 2021) fixing midpoint root and removing branches with less than 90 of bootstrap value. Secondly, viral intergenomic similarity between our phages and phages phylogenetically related was calculated for clustering in species and genera (95% and 70% thresholds respectively) using VIRIDIC (Moraru et al., 2020).

### 2.9 Phage-bacteria inhibition assay

The infectivity profile of phages was assessed at multiplicity of infection (MOI) 0.1, 1 and 10 using inhibition assays in liquid using a microplate reader (TECAN, Switzerland). The McFarland inoculation standards (0.5 ± 0.02) of each clinical isolate (~10^8^ UFC/ml) were prepared and the required volume of phage stock solution (10^8^ PFU/ml) was added to the MHB suspension to achieve MOI 0.1, 1 and 10 and a final volume of 200 µl containing 10 mM MgSO_4_ and 10 mM CaCl_2_. Samples were incubated using sterile MicroWell 96-well flat-bottom plate (ThermoFisher Scientific, USA) at 37°C with shaking orbital amplitude of 5 mm. The OD_595_ value of each well was measured every 5 min for 15 h.

### 2.10 Biofilm phage susceptibility test

The isolated phage effect on *K. pneumoniae* biofilm was determined using a modified methodology (Kropinski, 2018). Biofilm was formed by inoculating 100 µl of cation-adjusted Müeller-Hinton broth (CAMHB) containing 10^6^ CFU/ml of bacteria on the bottom of the wells of a 96-well flat-bottom plate (ThermoFisher Scientific, USA). The plate was statically incubated at 37°C for 18 h. Supernatant was removed and each well was filled with 200 µl of CAMHB containing different concentrations of phages in CAMHB supplemented with 10 mM CaCl_2_ and 10 mM MgSO_4_, ranging from 10^8^ to 10^5^ PFU/ml. The plate was statically incubated at 37°C for 20 h. After incubation, 100 µl of the supernatant from each well were aspirated and deposited in a new 96-well plate adding 10 μl of 5 mg/ml of 3-(4,5-dimethylthiazol-2-yl)-2,5-diphenyltetrazolium bromide (MTT) (Sigma-Aldrich, Germany) in sterile water. The plate was incubated under agitation (80–100 rpm) for 1 h at 37°C. The planktonically bacterial concentration derived from the biofilm was estimated by measuring the absorbance at 570 nm in a spectrophotometer. The remaining volume in each well was aspirated, and each well was washed one time with 200 μl of sterile saline. Then, 200 μl of TSB supplemented with 0.5 mg/ml of MTT were deposited in each well. The plate was incubated under agitation (80–100 rpm) for 1 h at 37°C. The viability of the bacteria embedded in the biofilm (biofilm viability) was estimated by measuring the absorbance at 570 nm in a plate reader. The experiments were performed in triplicate and six technical replicates per replicate.

### 2.11 Statistical analysis

Data distribution was evaluated using Shapiro-Wilk or Kolgomorov-Smirnov statistics. Descriptive statistics are cited as median and interquartile range (non-normal distribution) for each variable that were calculated. Non-parametric Mann-Whitney test considering equality of variances was used to compare two groups and non-parametric Kruskal-Wallis’s test was used to compare more than two groups. To determine the effect of temperature on the phage viability data was analyzed using linear regression. Bacteriophage inhibition of bacterial biofilm was analyzed by Dunn’s pairwise test with a Benjamini-Hochberg’s procedure. The possible relation between the bacterial biofilm or planktonic concentration and the concentration of phage was determined by using Spearman rank correlation coefficient. Significance level was established at α=0.05. All statistical analysis was performed using R (R Core Team, 2017) with R commander, except for linear regressions that were carried out using GraphPad Prism v.8 (GraphPad Prism, version 8.0.1); Windows Version by Software MacKiev ^©^ 2020–2018 GraphPad Software, LLC, USA) and STATA statistical software, release 11 (StataCorp, 2009, StataCorp LP, USA).

## 3 Results

### 3.1 F13 and F14 are stable at physiological conditions

**Figure 1:**
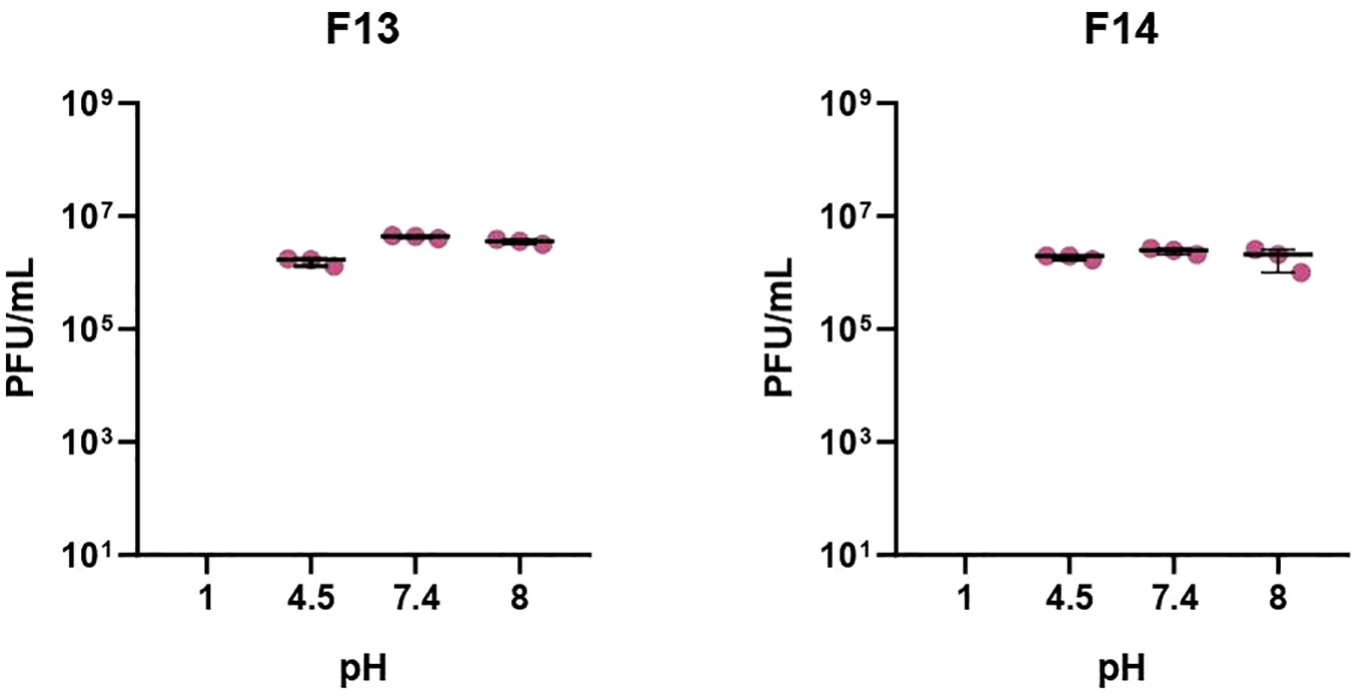
pH stability of bacteriophages. Incubation for 1h at pH of 1, 4.5, 7.4, and 8. Results are based on three repetitions. The bars represent the median and the interquartile range. “X” axis denotes limit detection of quantification (10 cfu/ml).

**Figure 2:**
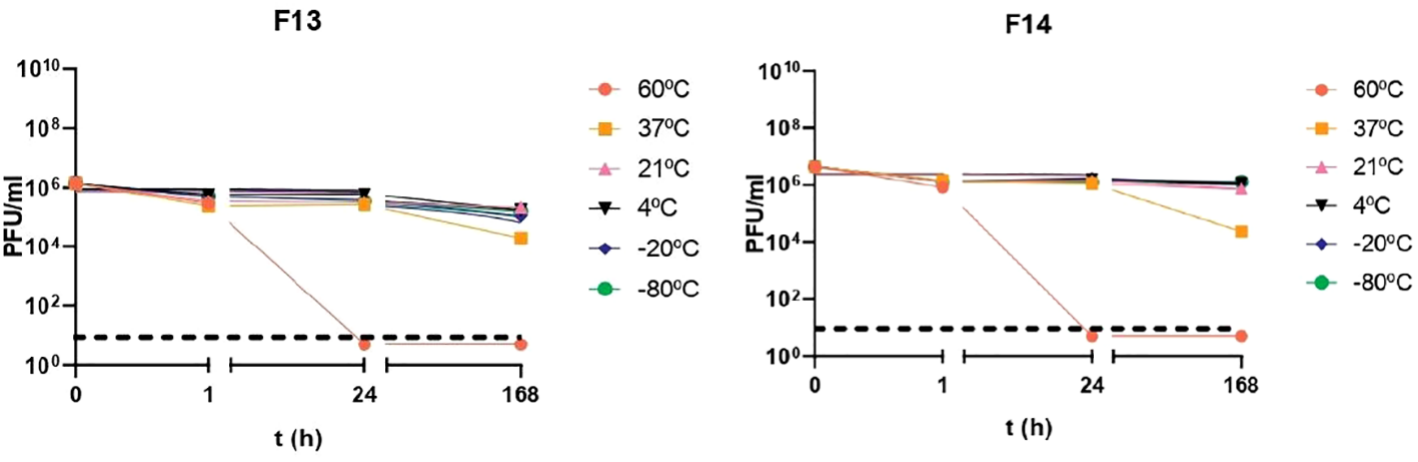
Temperature stability of bacteriophages. Incubation for 1 h, 24 h and 168 h at temperatures of -80°C, -20°C, 4°C, 21°C, 37°C and 60°C. Results are based on three repetitions. The bars represent the median and the interquartile range. Discontinued line denotes limit detection of quantification.

Phages were tested against a wide pH range after 1 h incubation to establish their activity under neutral, acid, and alkaline conditions (**Figure 1**). After 1 h incubation at pH 1, no plaques were detected in 100 μl of sample, suggesting that phages were inactivated at that pH (p-value<0.05). The rest of pH tested did not produce logarithmic reductions after 1 h of treatment for the two phages. Phages were also exposed to different temperatures: -80°C, -20°C, 4°C, 21°C and 37°C and 60°C (**Figure 2**). F13 and F14 were stable at -80°C, -20°C, 4°C and 21°C after seven days of exposition. A certain reduction of phage viability was observed after seven days at 37°C and the main reduction of viability was observed at 60°C after 24 h for both phages.

### 3.2 Determination of capsular type and sequence type for clinical strains

Prior the analysis of the host range of the phages, it was mandatory to study the diversity of the clinical strains. Results for the gene product *wzi* for the clinical isolates of *K. pneumoniae* are shown in **Supplementary Table 2**. The type strain ATCC 23357 showed a KL22 capsular type, KP5 showed a KL24 capsule, and the pandrug-resistant KP showed a KL3 capsule. Three clinical strains could not be defined. Regarding the sequence type (ST) of the clinical strains susceptible to phages, the most common sequence types were ST11, ST14 and ST15 (**Supplementary Table 2**).

### 3.3 F13 and F14 show the same host range

F13 and F14 phages showed the same infection pattern and lysed 19 (susceptible strains) out of 47 K*. pneumoniae* strains indicating an acceptable lytic activity (40.42%) (**Supplementary Table 3**). All tested strains were MDR *K. pneumoniae* strains (like KP5) including 3 extensively drug-resistant (XDR) strains.

### 3.4 Latent period and burst size for F13 and F14

**Figure 3:**
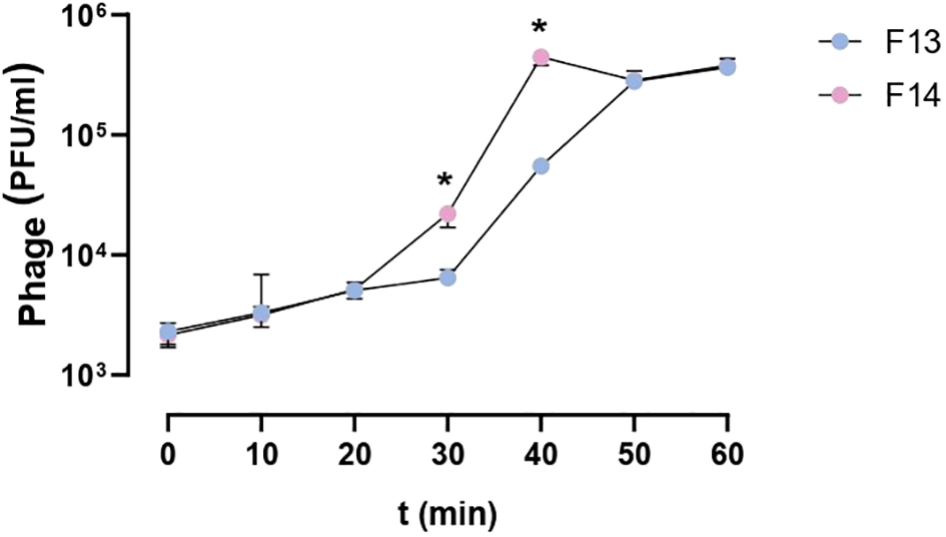
One-step growth curve of bacteriophages F13 and F14. The dependence of the bacteriophage titer on the incubation time in the phage-cell system with *K. pneumoniae* cells is shown. Results are based on four repetitions. The error bars represent the interquartile range. *p-value<0.05.

The replication cycle curve was determined by a one-step growth test. The latent period was 30 minutes for F13 and 20 minutes for phage F14. The burst size (average number of phage particles produced by one infected cell) were 56 plaque-forming units per infected cell for F13 (56 ± 12) and 87 plaque-forming units per infected cell for F14 (87 ± 13) (**Figure 3**). Therefore, the burst size of F14 was higher than of F13 with a significant statistical difference (p=0.004). The plaque-forming units per infected cell for F14 were higher compared to F13 at 30 min and 40 min (p=0.0286 for both time points) (**Figure 3**).

### 3.5 Both phages have a siphovirus morphotype

**Figure 4:**
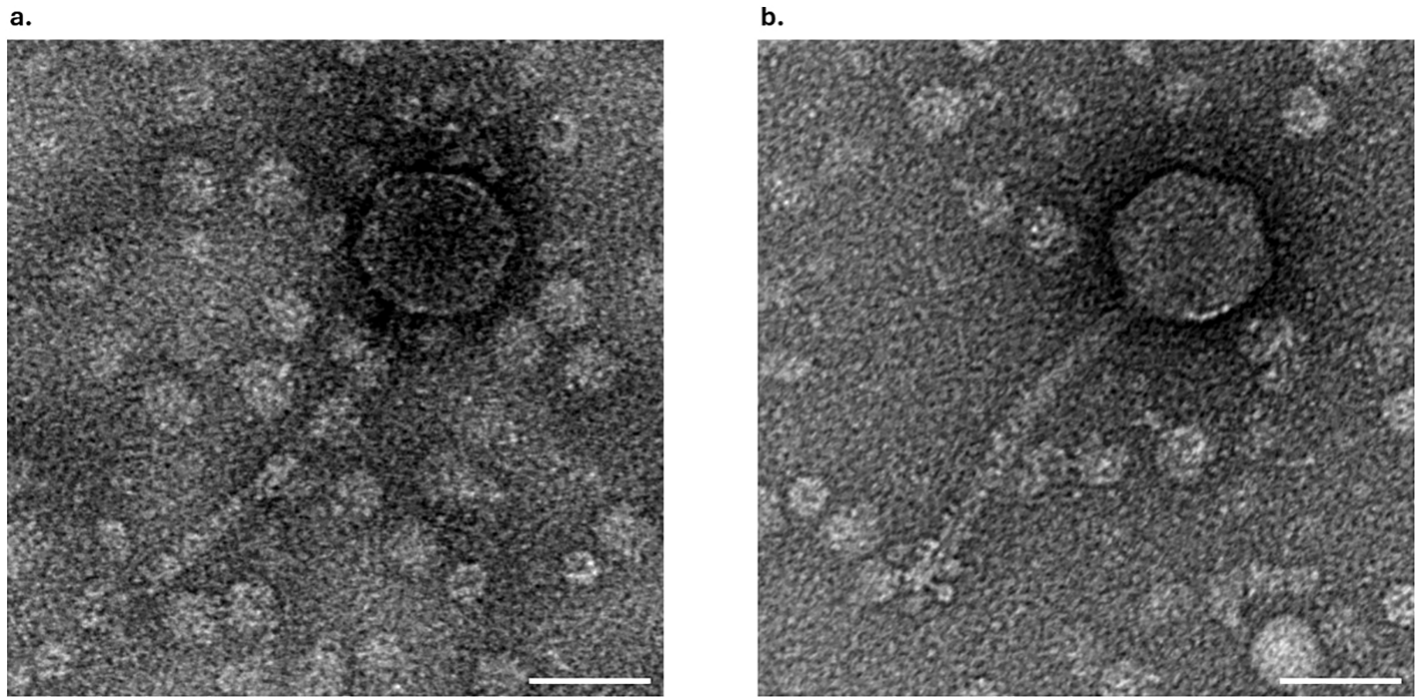
TEM analysis of bacteriophages. Electron micrograph of negatively stained F13 **(A)** and F14 **(B)** bacteriophages. Scale bars represent 50 nm.

The morphology of phage F13 and F14 was examined by transmission electron microscopy (TEM). Phage F13 had a 67 ± 5 nm (n = 30) head diameter and a 158 ± 11 nm (n = 21) tail length (**Figure 4A**). Phage F14 had a ≈65 ± 4 nm (n = 55) head diameter with a 156 ± 15 nm (n = 62) long tail (**Figure 4B**). Both phages had an icosahedral capsid and a long, flexible tail. These features correspond to the siphovirus morphotype (legacy family *Siphoviridae*). Therefore, phages F13 and F14 belong to the Caudoviricetes class of tailed bacteriophages with double-stranded DNA (dsDNA).

### 3.6 F13 is a novel *Webervirus* and F14 is a novel unclassified Caudoviricetes

Sequencing generated 130,913 clean reads for F13 and 594,563 clean reads for F14, whose *de novo* assembly with SPAdes (only-assembler mode) resulted in a contig of 49268 bp and 48812 bp respectively. Re-organization based on the long terminase subunit and correction of the assembly with Pilon generated a contig of 49,191 bp in the case of F13 and 48,735 bp in the case of F14, both with high coverage and a G + C content around 50% (**Table 1**).

**Table 1:** Summary of bacteriophages sequencing and genome annotations.

		vB_Kpn_F13 (Accession Number: PP341282)	vB_Kpn_F14 (Accession Number: PP341283)
**Size (bp)**		49191	48735
**GC content (%)**		50.43	48.16
**Average sequencing coverage**		361.47	1456.95
**Coding sequences**		84	95
**Most similar genome by BLAST**	**Name**	MW417503.1 *Klebsiella* phage LF20	NC_071149.1 *Klebsiella* phage vB_Kpn_ZCKp20p
	**Coverage (%)**	85	63
	**E-value**	0.0	0.0
	**Identity (%)**	91.65	94.83
**Taxonomy**	**Class**	Caudoviricetes	Caudoviricetes
	**Family**	Drexlerviridae	–
	**Genus**	Webervirus	–

General characteristics of phage genomes F13 and F14, including size, percentage of GC and coverage of genomes, predicted coding sequences by consensus gene calling, and most similar phage in the nucleotide database by BLASTn analysis.-: Taxonomic rank not determined by NCBI Taxonomy.

BLAST analysis against the nucleotide database found that 85% of F13’s genome was similar to genomes of phages belonging to *Webervirus* genus, included in the class *Caudoviricetes.* Regarding F14, 63% of its genome was similar to genomes previously described, all of them belonging to unclassified phages within the class *Caudoviricetes* (**Table 1**).

**Figure 5:**
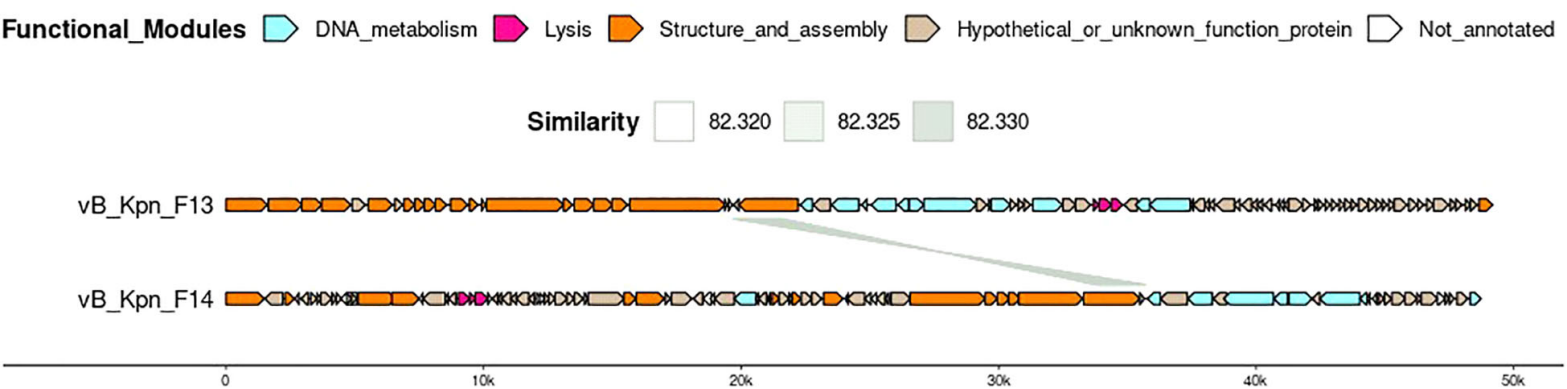
Genome organization and comparison of F13 and F14. Predicted coding sequences by the consensus of Glimmer, Prodigal and Phanotate are depicted using arrows with different colors depending on their function, predicted by BLASTp against the RefSeq viral protein database and mmseqs using phrogs profile database. Predicted coding sequences with no annotation remain white.

Structural annotation generated from the consensus of three gene callers (Glimmer, Prodigal and Phanotate) identified around 90 coding sequences in both genomes and most of them were functionally annotated by the combination of BLAST and mmseqs analysis (**Table 1**). Only 4 of the 84 coding sequences identified in F13 and 11 of the 95 in F14 remained unannotated (**Figure 5**).

The accession numbers of vB_Kpn_F13 and vb_Kpn_F14 are PP341282 and PP341283, respectively.

Genome organization of F13 was similar to other *Webervirus* phages previously described like the *Klebsiella* phage Npat (accession number OM938991.1) (Dunstan et al., 2023) while F14’s genome organization reminds of other unclassified viruses in the class *Caudoviricetes* like the *K. pneumoniae* phage vB_Kpn_ZC2 and the *Klebsiella* phage 6991 (accesion number NC_071156.1) (Fayez et al., 2023).

Interestingly, F13 and F14 shared a region of ~1870 bp encoding for a hypothetical protein and a tail fiber protein in opposite directions (similarity of 82%) (**Figure 5**). Safety analysis using PhageLeads (Yukgehnaish et al., 2022) confirmed F13 and F14 genomes do not contain any predicted temperate marker, virulence and antimicrobial resistance genes, which means that F13 and F14 are lytic.

**Figure 6:**
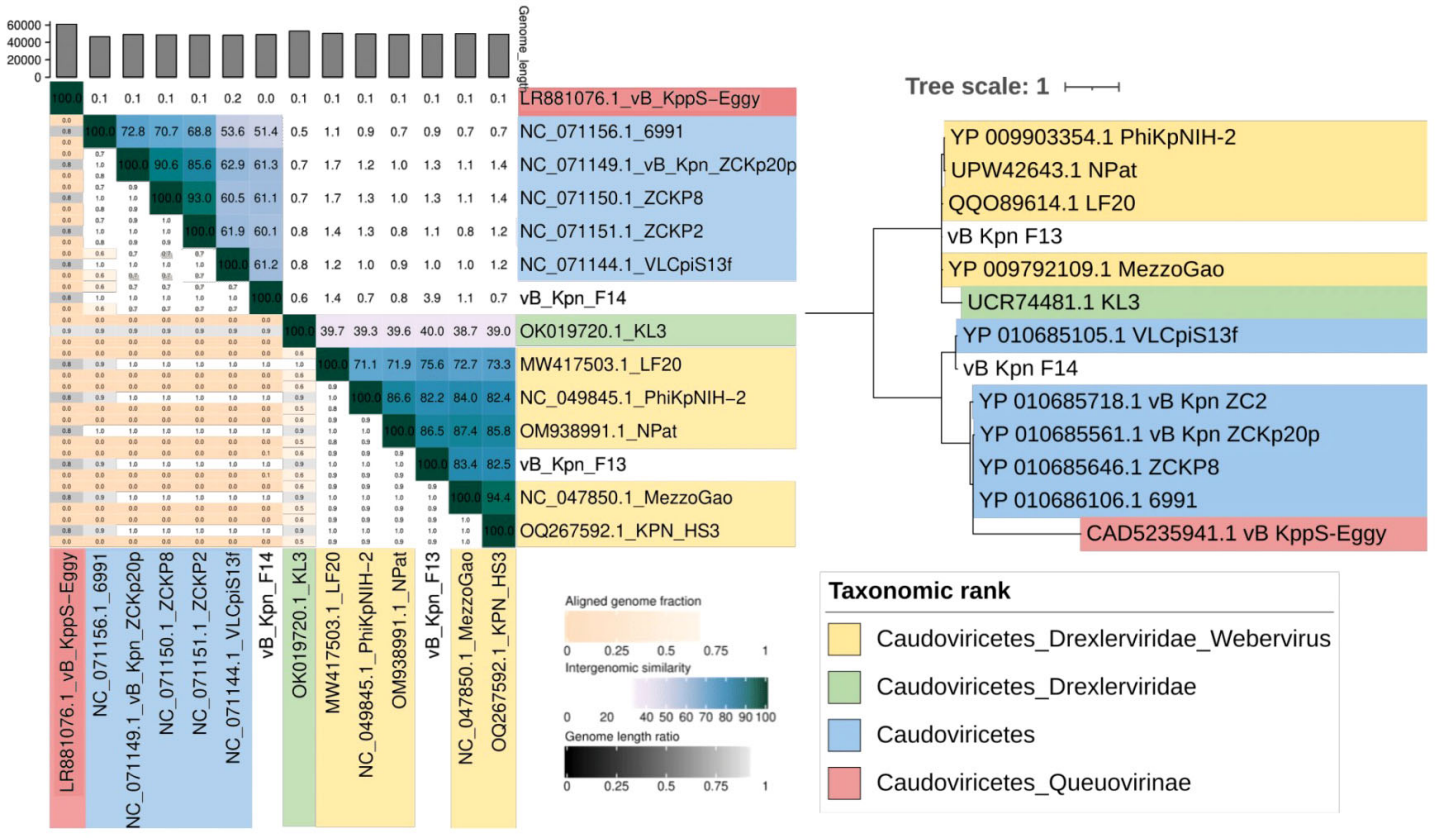
Taxonomy of bacteriophages. (Left) VIRIDIC clustering by intergenomic similarity (threshold of 95% and 70% for species and genera delimitation). (Right) Maximum likelihood phylogenetic tree based on the tail tape measure protein sequences of the phages isolated and phages belonging to different taxonomic ranks within the class Caudoviricetes.

VIRIDIC analysis clustered F13 with phages belonging to the *Webervirus* genus with intergenomic similarities ranging from 75,6 to 82,2% in all the cases while F14’s genome is more similar to unclassified phages among *Caudoviricetes* class (51,4–61,3% of intergenomic similarity) (**Figure 6**). Complementary, phylogenetic analysis using the amino acid sequence of the tail tape measure protein, a core protein of *Webervirus* genus (Maffei et al., 2021), provided a similar result with both phages grouping together with phages aforesaid (**Figure 6**).

### 3.7 The cocktail of F13 and F14 delays the planktonic bacterial growth modestly

**Figure 7:**
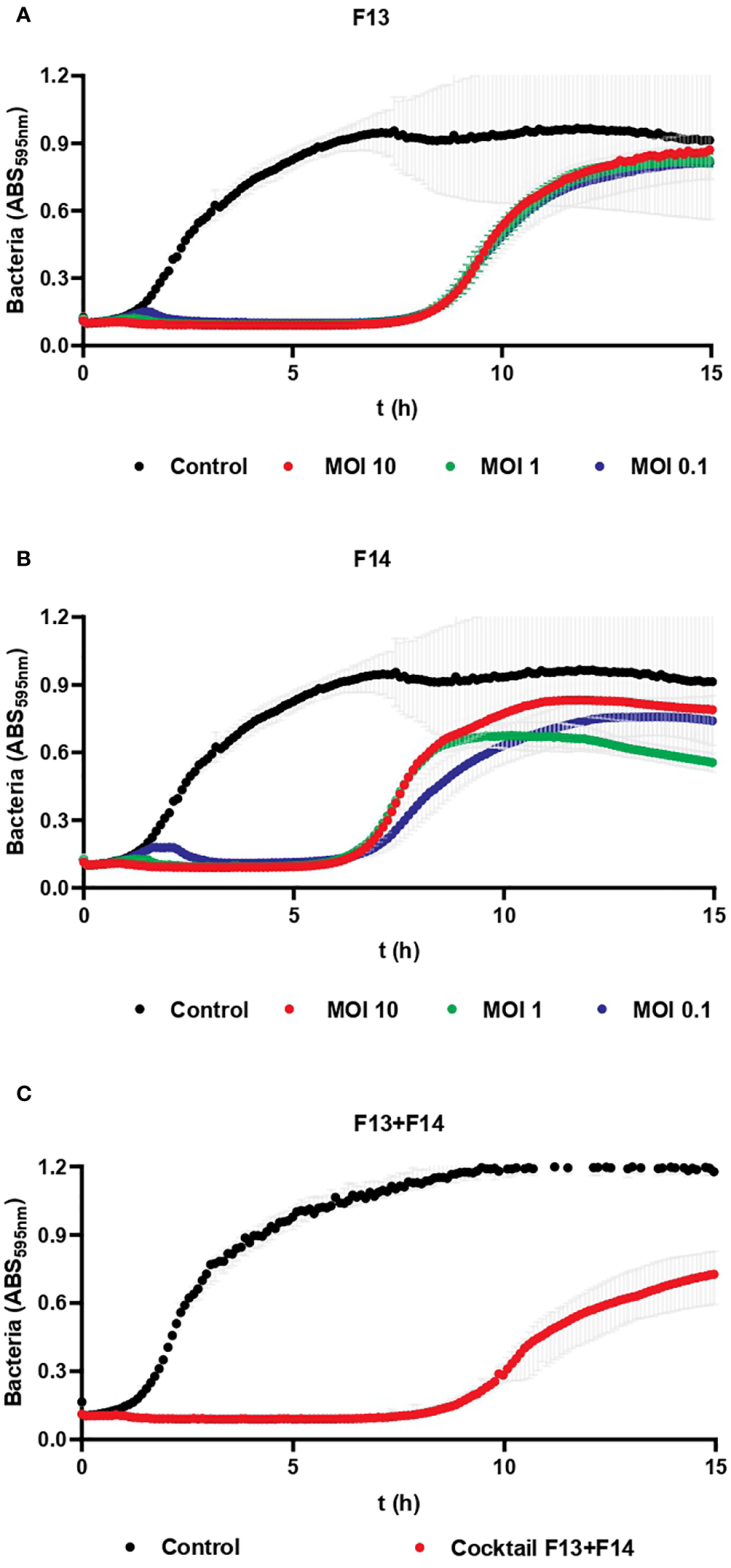
Bacteriophage inhibition assays against a clinical strain. **(A)** Inhibition of KP5 strain after treatment with F13 at MOI (10, 1 and 0.1). **(B)** Inhibition of KP5 strain after treatment with F14 at MOI (10, 1 and 0.1). **(C)** Inhibition of KP5 strain after treatment with a cocktail of F13 and F14 at MOI 10. Results are based on three repetitions. The grey bars represent the interquartile range.

Phage inhibition of KP5 was assessed every 5 min for 15 h when infected at MOI 10, 1 and 0.1 for F13 (**Figure 7A**), F14 (**Figure 7B**), and for a cocktail of both phages F13 and F14 (**Figure 7C**). The different MOIs of both phages had a similar effect over the bacterial growth with no significant differences between MOIs. Phages F13 and F14 alone were able to prevent bacterial planktonic growth for approximately 8 h and 7 h, respectively, while the cocktail inhibited the bacterial growth for 10 hours. Therefore, the combination use of F13 and 14 is more beneficial for bacterial growth than the use of a single phage only. Statistically significant differences were observed in bacterial growth of the three clinical isolates of KP5 according to the MOI of F13 and F14 F1Pa at 5, 10, and 15 h (**Supplementary Tables 4–9**).

### 3.8 F13 and F14 diminishes biofilm formation in a time-dependent concentration-dependent manner

Biofilm experiments studied the effect of phages against biofilm and against planktonic bacteria derived from biofilm. For a better comprehension of the obtained results a representative image was created (**Supplementary Figure 1**).

**Figure 8:**
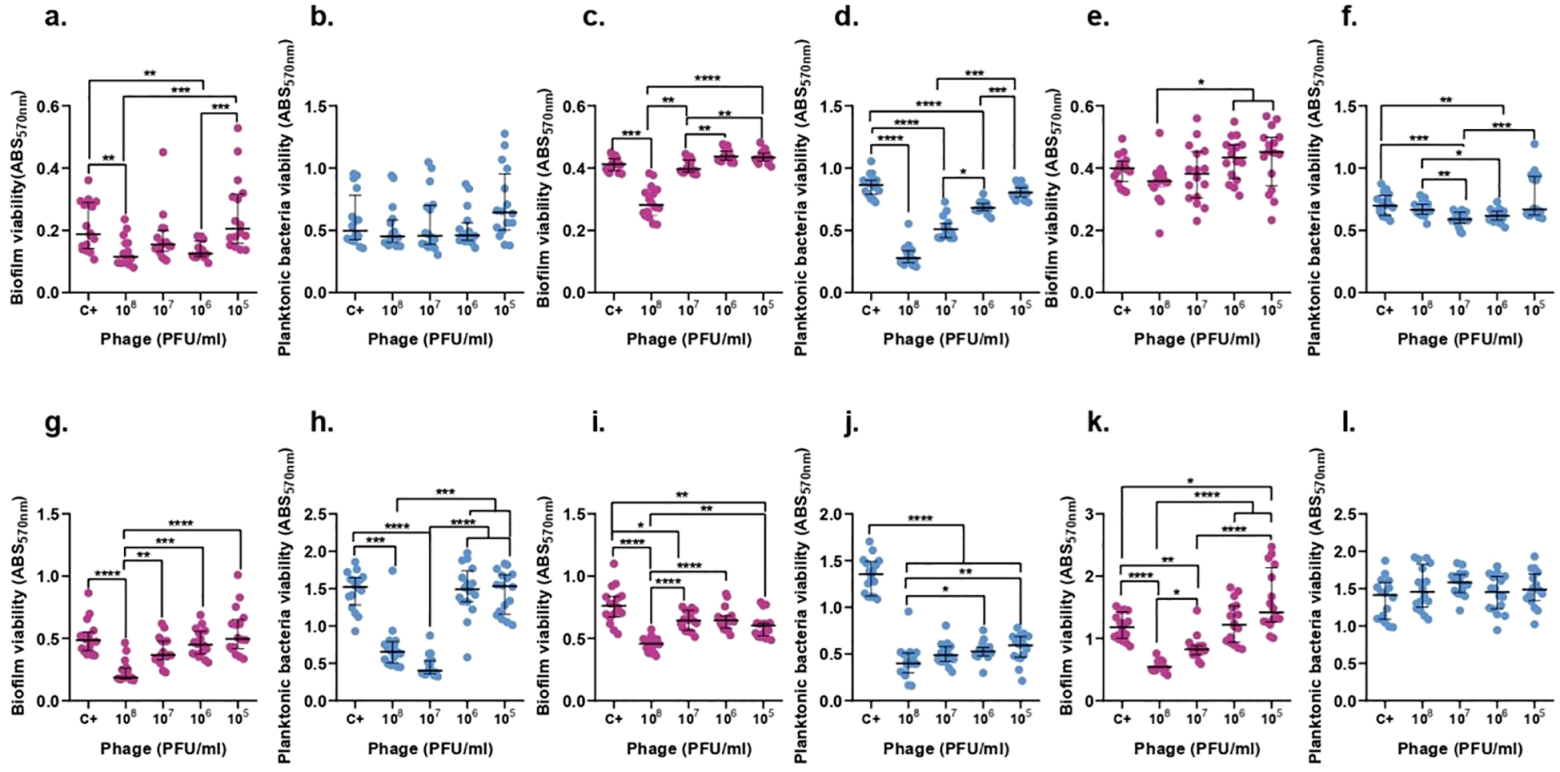
F13 effect on both *K. pneumoniae* biofilm (magenta) and planktonic bacteria originating from the biofilm (blue) at 6h (top row) and 24h (bottom row). ATCC 23357 **(A, B, G, H)**, KP5 **(C, D, I, J)**, and pandrug-resistant KP clinical strain **(E, F, K, L)**. Results are based on three repetitions. The bars represent the median and the interquartile range. *p-value<0.05, **p-value<0.01, ***p-value<0.001, ****p-value<0.0001 for Dunn’s test.

The concentration of ATCC 23357 of planktonic bacteria derived from biofilm did not show any significant decrease after 6 h of treatment in any concentration of F13, however, it was reduced by 75.5% after 24 h with 10^7^ PFU/ml of phages. Importantly, biofilm was decreased significantly with 10^8^ PFU/ml of F13 by 38.51% (p-value<0.05) after 6 h and 61.74% after 24 h (**Figure 8**).

**Figure 9:**
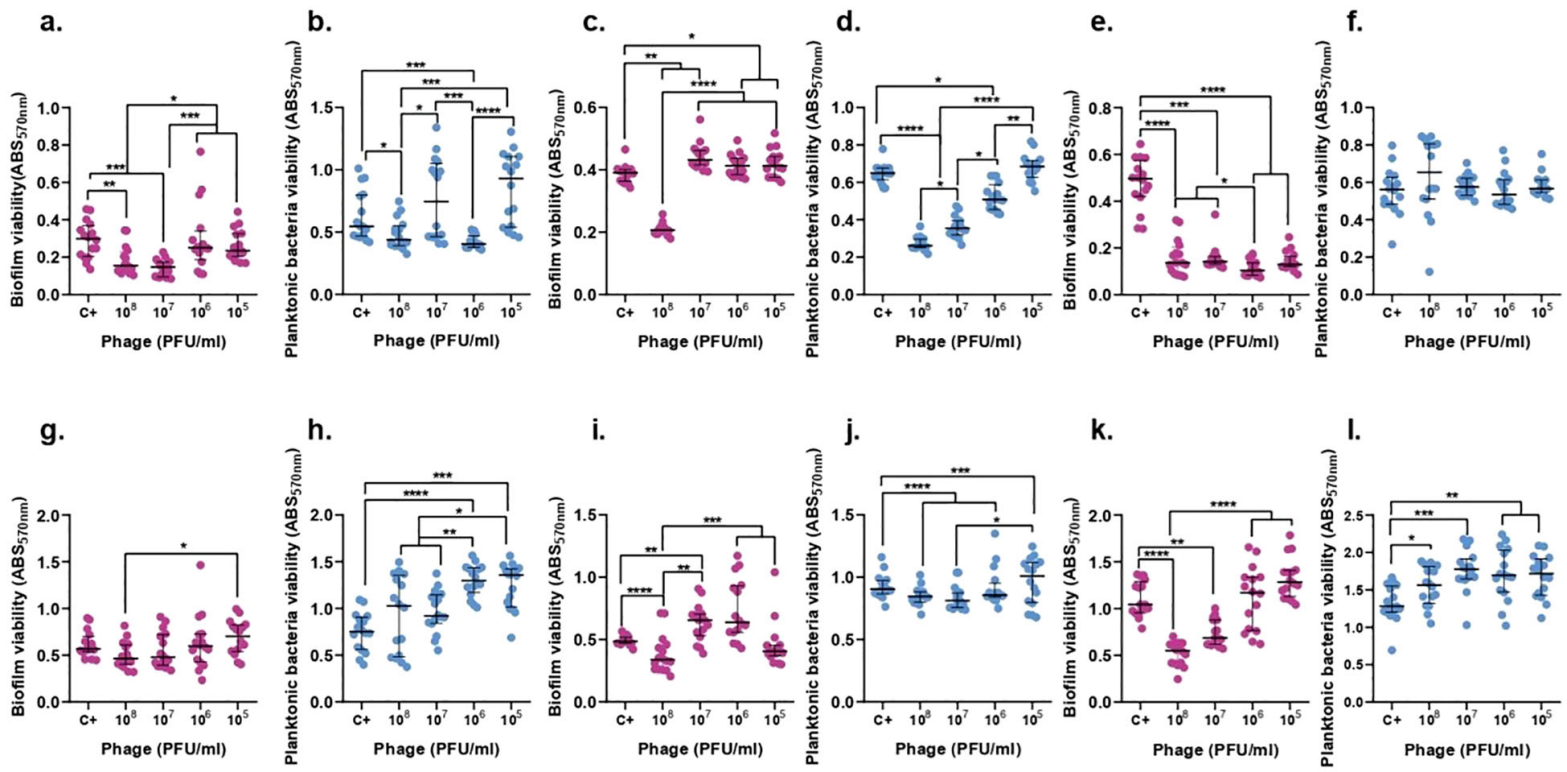
F14 effect on both K. pneumoniae biofilm (magenta) and planktonic bacteria originating from the biofilm (blue) at 6h (top row) and 24h (bottom row). ATCC 23357 **(A, B, G, H)**, KP5 **(C, D, I, J)**, and pandrug-resistant KP clinical strain **(E, F, K, L)**. Results are based on three repetitions. The bars represent the median and the interquartile range. *p-value<0.05, **p-value<0.01, ***p-value<0.001, ****p-value<0.0001 for Dunn’s test.

In case of phage F14, the biofilm of ATCC 23357 and the planktonic derived bacteria did show a significant decrease by 50.9% and 25.93% after 6 hours with 10^7^ and 10^6^ PFU/ml of F14 (p-value<0.05), respectively. Surprisingly, neither the biofilm nor planktonic bacteria derived from biofilm were significantly reduced after 24 h of treatment with F14 (**Figure 9**).

KP5 biofilm and planktonic bacteria derived from biofilm showed a significant decrease by 67.81% and 31.75% with a concentration of 10^8^ PFU/ml of F13 (p-value<0.05) after 6 h. Moreover, after 24 hours, the same quantity of phage was able to reduce the biofilm by 39.82% and the planktonic bacteria derived from biofilm by 71.25% (**Figure 8**).

F14 was able to decrease by 59.82% and 47.27% the biofilm of KP5 and planktonic bacteria derived from biofilm after 6 hours, respectively, with an inoculum of 10^8^ PFU/ml of F14 (p-value<0.05). After 24 hours, planktonic bacteria derived from biofilm were reduced by 64.99% with 10^7^ PFU/ml and biofilm was reduced by 41.31% with 10^8^ PFU/ml of F14 (**Figure 9**).

The viability of pandrug-resistant KP strain in planktonic bacteria derived from biofilm showed a significant decrease by 15.8% with 10^7^ PFU/ml of F13 (p-value<0.05), but no significant reduction in biofilm viability after 6 h. After 24 hours, only biofilm formation was reduced 56.32% with 10^8^ PFU/ml of F13 (**Figure 8**).

F14 was less effective than F13 against the pandrug-resistant KP strain in planktonic bacteria derived from biofilm showing no significant reduction after 6-h or 24-h treatments with 10^8^ PFU/ml (p-value<0.05). Importantly, biofilm formation did show a significant decrease by 73.83% with 10^5^ PFU/ml of F14 (p-value<0.05) after 6 hours and a reduction of 47.37% after 24 h of treatment with 10^8^ PFU/ml (**Figure 9**).

## 4 Discussion

In this study, we show two novel bacteriophages infecting carbapenem-resistant *K. pneumoniae* strains. Our susceptible bacterial strains showed different STs being ST11, ST14 and ST15 the most prevalent. ST11 has been reported worldwide, including in North America, South America, and most countries in Europe where it was first found in France (Andrade et al., 2011). In addition, many bacteria sensitive to our phages harbored a KL24 capsule, which is highly prevalent in Spain according to CARB-ES-19 Multicenter Study (Cañada-García et al., 2022). Furthermore, the KL24 serotype was recently reported to be frequently associated with ST15 (94). *K. pneumoniae* phages usually infect the target bacteria depending on the capsule serotype, suggesting that F13 and F14 are phages that could potentially infect a greater number of clinical strains.

The isolated phages were found to be stable at a range of pH 4.5–8 and unstable at pH 1, meaning that an oral administration would require protection with a coating. These data are similar with other studies characterizing the stability of two *Lastavirus* phages (LASTA and SJM3) against a pandrug-resistant *K. pneumoniae* strain that were inactivated in the extreme acidic environment of pH 2 and at temperatures above 68°C (Obradović et al., 2023). They have similar stability to previously reported *K. pneumoniae* phages P929 and P13 (Chen et al., 2022; Fang and Zong, 2022). Other studies have also shown phages that lost infectivity at pH below 3 and relatively heat stable with good phage activity after exposure to temperatures between 25°C and 45°C and phage activity was completely lost at temperatures above 65°C (Horgan et al., 2010).

The novel phages showed similar latency periods being 30 minutes for F13 and 20 minutes for F14. The burst size for F13 and F14 was 56 and 87 plaque-forming units per infected cell (PFU/cell), respectively, which is superior to other *K. pneumoniae* phages, such as Henu1 or phage BUCT556A (Drulis-Kawa et al., 2011; Kęsik-Szeloch et al., 2013; Tabassum et al., 2018; Teng et al., 2019; Shi et al., 2020; Zhang et al., 2020; Feng et al., 2021; Bai et al., 2022; Mulani et al., 2022; Zaki et al., 2023) indicating that the new isolated phages have a strong lysis property. Other studies have shown a range of results for burst size and latency period for *K. pneumoniae* phages which suggest the variability and specificity of each isolated phage (Morozova et al., 2019; Obradović et al., 2023).

The electron microscopy images and genomic studies showed that the novel phages belonged to the siphovirus morphotype in the class *Caudoviricetes* (Guerra et al., 2022). F13 belongs to the genus *Webervirus*, family *Drexlerviridae.* Its morphology and dimensions are comparable to those of other phages in the genus and its genome is 85% homologous to them (Ackermann, 2009). F14 morphology and dimensions are also similar to its closest related phage, the *Klebsiella* phage vB_Kpn_ZCKp20p (Zaki et al., 2023). F14 shares 63% similarity with unclassified phages from the *Caudoviricetes* class, consequently, it most likely represents a new family within other unclassified bacteriophages.

The ability to form biofilms is a key virulence characteristic of *K. pneumoniae* (Guerra et al., 2022). Our results showed two different concentration-dependent effects of phages on *K. pneumoniae* biofilms, (i) an inhibitory effect at high concentrations, and (ii) a dispersive effect at low concentrations, or at any concentration. Though the dispersive effect is a known bacterial response against bacteriophages, the key point is that the presence of F13 or F14 would trigger the switch from biofilm to the planktonic state, which is more amenable to antibiotic treatment. Thanks to the depolymerases on their tail spikes, phages are capable of degrading biofilm by local hydrolyzation, and, in case of synergy, antibiotics could have access to deeper zones into the biofilm.

Phage-resistant mutants appear commonly when a single phage is used to treat bacteria. To overcome this problem, phage therapy usually uses phage cocktails, but it is not clear how many phages should be combined. Potential drawbacks of using multiple phages together, such as competition between phages, could take place and would be undesirable. *In vitro* studies should be performed to avoid inefficiency in the patient. F13 combined with F14 showed a delay in liquid growth of the target bacteria from 7 to 10 hours and a decrease of the bacterial growth afterwards. However, to analyze the real effect of the cocktail compared to the individual use of F13 and F14, biofilm assays and animal assays should be performed as well.

All these results show that the novel isolated phages, vB_Kpn_F13 and vB_Kpn_F14 are stable at physiological conditions, exhibit good lytic activity, present a broad-spectrum range as well as inhibitory effect against carbapenem-resistant strains in solid, liquid and biofilm growth. The results of inhibition in solid, liquid and biofilm are quite similar for both phages, so we cannot be certain if F14 is a better candidate than F13 or the contrary. Both bacteriophages are potential antimicrobials to be used as an alternative therapy and to conduct further studies *in vivo*.

There are some limitations to consider in phage therapy research. For *in vitro* studies we always know the number of cfu/ml of bacteria and pfu/ml of phages at the beginning of the experiment, however, in real infections we do not know the multiplicity of infection of phages we are adding. Moreover, the immunological effect of the patient could reduce even more the MOI. In addition, infections related with biofilms include polymicrobial infections which need a specific study to conclude about the efficiency in this situation. In general, the *in vitro* results show the effect in optimal conditions, *in vivo* experiments should be performed to know if the pharmacokinetics and pharmacodynamics of phages are optimal to reach the specific site of infection with a good concentration.

## Data Availability

The data presented in the study are deposited in the GenBank database, the accession numbers of vB_Kpn_F13 and vb_Kpn_F14 are PP341282 and PP341283, respectively.
