# Supplementary material for: Isolation and characterization of two novel bacteriophages against carbapenem-resistant Klebsiella pneumoniae
Source: Front Cell Infect Microbiol. 2024 Aug 29;14:1421724. doi: 10.3389/fcimb.2024.1421724 (PMC11390652; doi:10.3389/fcimb.2024.1421724)

**Supplementary Figures and Tables**

**Supplementary Table 1.** Antibiogram of K. pneumoniae strains

| **Antibiotic** | **ATCC 23357 (MIC)** | **KP5 (MIC)** | **Pandrug-resistant KP (MIC)** |  |
| --- | --- | --- | --- | --- |
| Amikacin | S (4) | S (4) | S (<2) |  |
| Ampicilin | R (<32) | R (>32) | R (>32) |  |
| Cefotaxime | S (<0.25) | R (64) | R (>64) |  |
| Cefoxitine | S (4) | S (8) | R (32) |  |
| Ceftazidime | S (0.5) | R (>64) | R (>34) |  |
| Gentamycin | S (<1) | S (<1) | R (>16) |  |
| Tobramycin | S (<1) | R (8) | R (8) |  |
| Imipenem | S (<0.25) | R (>16) | R (>16) |  |
| Cefuroxime | S (8) | R (>64) | R (>64) |  |
| Amoxicilin-clavulanic acid | S (8) | R (>32) | R (>32) |  |
| Ciprofloxacin | S (<0.25) | R (>4) | R (1) |  |
| Fosfomycin | R (64) | R (>256) | R (>256) |  |
| Co-trimoxazole | S (<20) | R (>320) | R (>320) |  |
| Cefuroxime-axetil | S (8) | R (>64) | R (>64) |  |
| Cefepime | S (<0.12) | R (>32) | R (>64) |  |
| Colistin | S (0.125) | S (0.125) | S (0.5) |  |
| Ertapenem | S (<0.12) | R (>8) | R |  |
| BSBL | Negative | Positive | Negative |  |
| Ceftazidime/avibactam | S (0.75) | S (0.75) | R (>256) |  |
| Nadilixic acid | S (4) | R (>32) | R (>32) |  |
| Nitrofuration | S (<16) | R (128) | R (128) |  |

**Supplementary Table 1.** Antibiogram of K. pneumoniae strains used in this study. MIC: minimum inhibitory concentration. MIC determined by E-test®. BSBL: Broad-spectrum β-lactamase. S: susceptible. R: Resistant. I: intermediate.

**Supplementary Table 2.** Sequence type and capsular type of bacterial strains susceptible to phages F13 and F14

| **Strain** | **Sequence Type (ST)** | **Predicted serotype** |  |
| --- | --- | --- | --- |
| **KP1** | ST14 | KL24 |  |
| **KP5** | ST15 | KL24 |  |
| **KP6** | ST11 | KL24 |  |
| **KP8** | ST326 | KL24 |  |
| **KP9** | ST2237 | KL24 |  |
| **KP18** | ST15 | KL24 |  |
| **KP21** | ST15 | KL24 |  |
| **KP22** | ST1068 | KL24 |  |
| **KP26** | ST14 | KL24 |  |
| **KP29** | ST5031 | KL24 |  |
| **KP32** | ST175 | KL24 |  |
| **KP52** | ST29 | KL112 |  |
| **KP74**  **KP 4940164**  **ATCC 23357** | ST15  ST14  ST11 | KL24  KL3  KL22 |  |
| **A7** | ST26 | KL24 |  |
| **A22** | ST204 | ND |  |
| **A28** | ST318 | ND |  |
| **A29** | ST1 | ND |  |

**Supplementary Table 2**. Sequence type (ST) and capsular type (KL) analysis over the susceptible clinical strains to phages F13 and F14. ND: not defined.

**Supplementary Table 3.** Summary of bacteriophages host range

| **Strain** | **Origin** | **MDR/XDR** | **F13** | **F14** |
| --- | --- | --- | --- | --- |
| **KP1** | Pharynx | MDR | **+** | **+** |
| **KP2** | Joint | MDR | **-** | **-** |
| **KP5** | Urine | MDR | **+** | **+** |
| **KP6** | Unknown | MDR | **+** | **+** |
| **KP8** | Urine | MDR | **+** | **+** |
| **KP9** | Urine | MDR | **+** | **+** |
| **KP10** | Unknown | MDR | **-** | **-** |
| **KP18** | Urine | MDR | **+** | **+** |
| **KP19** | Bronchial | MDR | **-** | **-** |
| **KP20** | Urine | MDR | **-** | **-** |
| **KP21** | Urine | MDR | **+** | **+** |
| **KP22** | Urine | MDR | **+** | **+** |
| **KP26** | Urine | MDR | **+** | **+** |
| **KP27** | Urine | MDR | **-** | **-** |
| **KP28** | Urine | MDR | **-** | **-** |
| **KP29** | Urine | MDR | **+** | **+** |
| **KP32** | Urine | MDR | **+** | **+** |
| **KP50** | Urine | MDR | **-** | **-** |
| **KP51** | Pharynx | MDR | **-** | **-** |
| **KP52** | Urine | MDR | **+** | **+** |
| **KP74** | Urine | MDR | **+** | **+** |
| **A7** | Wound | MDR | **+** | **+** |
| **A12** | Wound | MDR | **-** | **-** |
| **A14** | Perianal | MDR | **-** | **-** |
| **A15** | Perianal | MDR | **-** | **-** |
| **A17** | Urine | MDR | **-** | **-** |
| **A19** | Urine | MDR | **-** | **-** |
| **A20** | Perianal | MDR | **-** | **-** |
| **A21** | Nasal swab | MDR | **-** | **-** |
| **A22** | Nasal swab | MDR | **+** | **+** |
| **A23** | Perianal | MDR | **-** | **-** |
| **A24** | Bronchial | MDR | **-** | **-** |
| **A25** | Bronchial | MDR | **-** | **-** |
| **A26** | Perianal | MDR | **-** | **-** |
| **A27** | Perianal | MDR | **-** | **-** |
| **A28** | Blood | MDR | **+** | **+** |
| **A29** | Urine | MDR | **+** | **+** |
| **A30** | Urine | MDR | **-** | **-** |
| **A31** | Perianal | MDR | **-** | **-** |
| **A32** | Perianal | MDR | **-** | **-** |
| **A33** | Perianal | MDR | **-** | **-** |
| **A34** | Urine | MDR | **-** | **-** |
| **ATCC 23357** | Type strain | MDR | **+** | **+** |
| **KP 4911574** | Bronchial | XDR | **-** | **-** |
| **KP 4912237** | Bronchial | XDR | **-** | **-** |
| **KP 4940164** | Bronchial | MDR | **+** | **+** |
| **KP 4911623** | Bronchial | XDR | **-** | **-** |
|  |  |  |  |  |

**Supplementary Table 3.** Summary of K. pneumoniae bacteriophages host range. Based on lysis of bacteria, strains were differentiated into two categories: susceptible (+) or non-susceptible (-) to phages. Results are based on four repetitions. MDR: multidrug-resistant; XDR: extensively drug-resistant.

**Supplementary Table 4.** Pairwise comparison of Dunn's test between the absorbance of *K. pneumoniae* clinical isolate KP5 and the different multiplicity of infection (MOIs) of bacteriophage F13 at 5 h.

|  | **Control** | **MOI 10** | **MOI 1** |
| --- | --- | --- | --- |
| **MOI 0.1** | 1.423024  0.1160 |  |  |
| **MOI 1** | 2.371708  0.0266 | 0.821583  0.2057 |  |
| **MOI 10** | 3.320391  0.0027* | 1.643167  0.1003 | 0.821583  0.2468 |

**Supplementary Table 5.** Pairwise comparison of Dunn's test between the absorbance of *K. pneumoniae* clinical isolate KP5 and the different multiplicity of infection (MOIs) of bacteriophage F13 at 10 h.

|  | **Control** | **MOI 10** | **MOI 1** |
| --- | --- | --- | --- |
| **MOI 0.1** | 2.266298  0.0351 |  |  |
| **MOI 1** | 3.320391  0.0027* | 0.912870  0.2168 |  |
| **MOI 10** | 1.528434  0.0948 | -0.639009  0.2614 | -1.551880  0.1207 |

**Supplementary Table 6.** Pairwise comparison of Dunn's test between the absorbance of *K. pneumoniae* clinical isolate KP5 and the different multiplicity of infection (MOIs) of bacteriophage F13 at 15 h.

|  | **Control** | **MOI 10** | **MOI 1** |
| --- | --- | --- | --- |
| **MOI 0.1** | 2.582526  0.0147* |  |  |
| **MOI 1** | 3.109573  0.0056* | 0.456435  0.3240 |  |
| **MOI 10** | 1.423024  0.1160 | -1.004158  0.1892 | -1.460593  0.1441 |

**Supplementary Table 7.** Pairwise comparison of Dunn's test between the absorbance of *K. pneumoniae* clinical isolate KP5 and the different multiplicity of infection (MOIs) of bacteriophage F14 at 5 h.

|  | **Control** | **MOI 10** | **MOI 1** |
| --- | --- | --- | --- |
| **MOI 0.1** | 1.423024  0.1160 |  |  |
| **MOI 1** | 2.477117  0.0199* | 0.912870  0.2168 |  |
| **MOI 10** | 3.214982  0.0039* | 1.551880  0.1207 | 0.639009  0.2614 |

**Supplementary Table 8.** Pairwise comparison of Dunn's test between the absorbance of *K. pneumoniae* clinical isolate KP5 and the different multiplicity of infection (MOIs) of bacteriophage F14 at 10 h.

|  | **Control** | **MOI 10** | **MOI 1** |
| --- | --- | --- | --- |
| **MOI 0.1** | 1.423024  0.1160 |  |  |
| **MOI 1** | 3.109573  0.0056* | 1.460593  0.1441 |  |
| **MOI 10** | 2.582526  0.0147* | 1.004158  0.1892 | -0.456435  0.3240 |

**Supplementary Table 9.** Pairwise comparison of Dunn's test between the absorbance of *K. pneumoniae* clinical isolate KP5 and the different multiplicity of infection (MOIs) of bacteriophage F14 at 15 h.

|  | **Control** | **MOI 10** | **MOI 1** |
| --- | --- | --- | --- |
| **MOI 0.1** | 3.004163  0.0080* |  |  |
| **MOI 1** | 2.477117  0.0199* | -0.456435  0.3240 |  |
| **MOI 10** | 1.633843  0.1023 | -1.186732  0.1765 | -0.730296  0.2791 |

**Supplementary Figure 1.** Representative image of the effect of F14 on planktonic bacteria derived from biofilm and the biofilm at the bottom of the wells of 96-well plates of the KP5 strain after 6 and 24 hours of treatment and staining with dimethylthiazol-2-yl-2,5-diphenyltetrazolium bromide (MTT). PFU: plate-forming units.


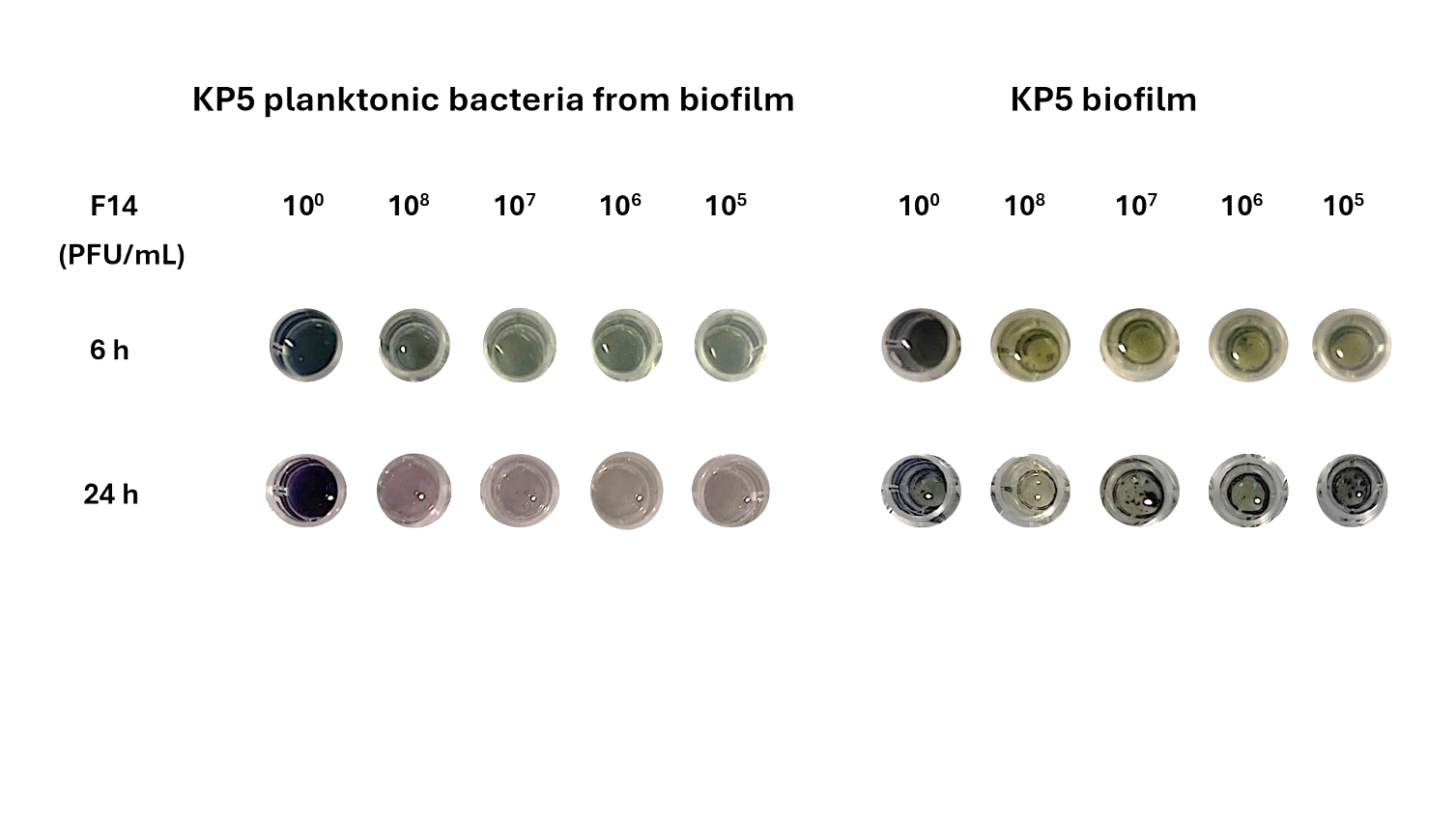

Supplement: Supplementary file 1 [file Table1.docx]
